# The long-term changing patterns of suicide mortality in China from 1987 to 2020: continuing urban-rural disparity

**DOI:** 10.1186/s12889-024-18743-z

**Published:** 2024-05-09

**Authors:** Yu Wu, Binbin Su, Panliang Zhong, Yiran Wang, Yueqin Huang, Xiaoying Zheng

**Affiliations:** 1 Department of Population Health and Aging Science, School of Population Medicine and Public Health, Chinese Academy of Medical Sciences & Peking Union Medical College, No. 31, Road 3rd, Bei-Ji-Ge, Dongcheng District, Beijing, 100730 China; 2 APEC Health Science Academy (HeSAY), Peking University, Beijing, China; 3 NHC Key Laboratory of Mental Health (Peking University), Peking University Sixth Hospital, Peking University Institute of Mental Health, National Clinical Research Center for Mental Disorders (Peking University Sixth Hospital), Beijing, 100191 China

**Keywords:** Suicide, Mortality, Urban-rural disparity, Age disparity, Gender reversal, China

## Abstract

**Background:**

Over the past three decades, China has experienced significant changes in urban-rural, gender, and age-specific suicide mortality patterns. This study aimed to investigate the long-term trends in suicide mortality in China from 1987 to 2020.

**Methods:**

Suicide mortality data were obtained from China’s National Health Commission. Joinpoint regression analysis was used to examine changes in trends and age-period-cohort modeling to estimate age, period, and cohort effects on suicide mortality from 1987 to 2020. Net drift, local drift, longitudinal age curves, and period relative risks were also calculated.

**Results:**

Crude and age-standardized suicide mortality in China showed continuing downward trends from 1987 to 2020, with a more pronounced decrease in rural areas (net drift = -7.07%, *p*<0.01) compared to urban areas (net drift = -3.41%, *p*<0.01). The decline curve of urban areas could be divided into three substages. Period and cohort effects were more prominent in rural areas. Suicide risk was highest among individuals aged 20–24 and gradually increased after age 60. Females, particularly those of childbearing age, had higher suicide risk than males, with a reversal observed after age 50. This gender reversal showed distinct patterns in urban and rural areas, with a widening gap in urban areas and a relatively stable gap in rural areas.

**Conclusions:**

Suicide mortality in China has consistently declined over the past three decades. However, disparities in age, gender, and urban-rural settings persist, with new patterns emerging. Targeted suicide prevention programs are urgently needed for high-risk groups, including females of childbearing age and the elderly, and to address the slower decrease and reversing urban-rural gender trends.

**Supplementary Information:**

The online version contains supplementary material available at 10.1186/s12889-024-18743-z.

## Introduction

Suicide is one of the leading causes of death worldwide, impacting all ages, genders, and regions worldwide [1]. According to the World Health Organization, more than 700,000 people worldwide die by suicide yearly [2]. From a global perspective, suicide mortality has been rising in recent years [1], which requires urgent attention and targeted prevention.

China used to be one of the nations with relatively high suicide mortality in the world, and there were significant urban-rural, gender, and age disparities in prior suicide mortality in China [3, 4]. Studies by Phillips et al. approximated the suicide mortality rate in China between 1995 and 1999 to be 23 per 100,000 [4]. The suicide mortality rate in rural regions was three times that of urban areas, while the mortality rate was found to be 25% higher in females than males [4]. Considering the scarcity of suicide research at that time, the alarmingly high suicide mortality and the unique pattern drew substantial attention. In the subsequent decades, China has undergone profound social changes, including economic growth, increased urbanization, an aging populace, and improved management of lethal substances [5]. Prior studies have documented a considerable reduction in overall suicide mortality rates in China over recent decades [6–10]. Shifts in suicide mortality patterns in relation to urban-rural, gender, and age demographics have also been decidedly dramatic [11]. For example, based on joinpoint regression analyses, Jiang et al. revealed a significant decrease in suicide mortality rates in China from 2002 to 2015, and uncovered a reversal in suicide mortality trends for males and females post-2006 [6]. By examining suicide mortality data encompassing 33 Chinese provinces, Zhang et al. found a 65% reduction in suicide mortality in China from 1990 to 2017. Also, the male-to-female suicide ratio shifted from 0.88 to 1.56 during this period [12]. Drawing on data from the China Health Statistics Yearbook, Liu et al. determined that urban residents and women experienced greater reductions in suicide mortality compared with rural inhabitants and men, respectively [10].

Previous studies have provided a reference for data on suicide research in China. However, an expansive, thorough examination of lifespan suicide risk variation, period and cohort suicide risk, and potential underlying causes has yet to be conducted. Additionally, previous suicide mortality trend investigations have been restricted to relatively short intervals, with a lack of assessments spanning periods longer than 30 years. It is unclear whether suicide mortality and the epidemiological distribution of suicide mortality rates in China have changed. Therefore, this study will employ Joinpoint regression analysis and an age-period-cohort model to evaluate long-term suicide mortality patterns from 1987 to 2020, using data sourced from the National Health Commission’s death registration system in China. The findings would provide certain reference for understanding suicide epidemiology in China and proposing suicide prevention strategies.

## Methods

### Data source

Data regarding age-specific suicide mortality from 1987 to 2020 was primarily obtained from the death registration system of the National Health Commission in China. The mortality records from four administrative organizations were integrated after the quality control of IDs and duplication removal. Administrative organizations mainly include: (1) the Department of Health, providing both the death medical certificate information and total population data; (2) the Department of Public Security, contributing the registered permanent residence cancellation data; (3) the Department of Civil Affairs, offering the cremation records; and (4) the Department of Social Security, supplying the social security termination information. Suicide was coded according to the International Classification of Diseases (ICD), the 9th Revision (ICD-9) operated before 2002, and ICD-10 thereafter (ICD-9: E950–E959, ICD-10: X60-X84). Due to the potential stigma associated with suicide, this dataset encounters similar underreporting issues as the majority of suicide mortality reporting data [4, 10, 13, 14]. Based on this data, previous studies have explored mortality patterns of various diseases such as mental disorders and respiratory diseases, as well as urban-rural life expectancy differences [13, 15, 16].

### Statistical analysis

Due to the exceedingly low incidence of suicide mortality in children under 10 years of age, this demographic was excluded from the scope of this study. Age-standardized mortality rates per 100,000 males and females by residence were calculated using the direct method based on the World Standard Population [17]. We utilized the National Cancer Institute’s Surveillance Research Program’s Joinpoint Regression Software, Version 4.9.1.0, to perform Joinpoint regression analysis, allowing us to evaluate how annual mortality rates alter and to pinpoint significant breakpoints [18, 19].

An age-period-cohort model was used to address the issue that the age effects, period effects and cohort effects were entirely correlated and develop independent effect estimates of age, period, and birth cohort on suicide mortality in China [20, 21]. In the age-period-cohort model, net drift represents the log-linear trend by period and cohort for the whole population, and local drift represents the log-linear trend by period and cohort for each age group [20, 22]. The model results showed a longitudinal age curve, period-relative, and cohort-relative risks. The longitudinal age curve is the rate curve of specific age groups in the reference cohort after adjusting for period effects. To avoid too many lines while reading a graph, we calculated the mortality and population data into consecutive 5-year periods from 1987 to 2020. The longitudinal age curve used successive 5-year age intervals from age 0–4 to age 85 or above among both urban and rural Chinese residents. The period-relative risks are the ratio of age-standardized rates in each period in the reference cohort, with the 2001–2005 survey year as the reference period group. The cohort-relative risks refer to the ratio of age-standardized and period-standardized mortality in 21 consecutive cohorts, including individuals born from 1906–1910 to 2006–2010, with the birth cohort of 1961–1965 as the reference group. We obtained estimated parameters from the United States National Cancer Institute’s age-period-cohort web-based tool [23]. The Wald χ^2^ test was used, and all statistical tests were two-sided for testing the significance of the estimable parameters and functions.

To maximize mitigation of the inherent limitations of the data used in this study and validate the robustness of the obtained results, we conducted the following sensitivity analyses: Firstly, using Global burden of disease (GBD) data, we performed similar analyses to compare patterns in suicide mortality rates under two different databases [24–26]. Secondly, to address the issues of disease coding changes in 2002 and urban-rural definition changes in 2005, we replaced the original data with the averages of mortality data from two years before and after 2002 and 2005, respectively [27], and conducted the same analysis to compare the patterns of suicide mortality rates before and after imputation. Thirdly, to adjust for the potential impact of underreporting of suicide deaths on our results, we employed the suicide underreporting rate adjustment method proposed by Li and Yip [14], which is also based on data from the National Health Commission in China. Due to the absence of undetermined deaths in the data from the National Health Commission in China, this method used accidental death rates (including drowning deaths, fall deaths, poisoning deaths, and other accidental deaths) to assess the underreporting of suicide by place (urban/rural), gender, and age in China [14]. It innovatively addressed the misclassification issue surrounding suicide deaths [14]. Based on the adjusted underreporting rates obtained, we conducted analyses similar to those in this study.

## Results

### Long-term trends in suicide mortality over the period 1987–2020

**Fig. 1:**
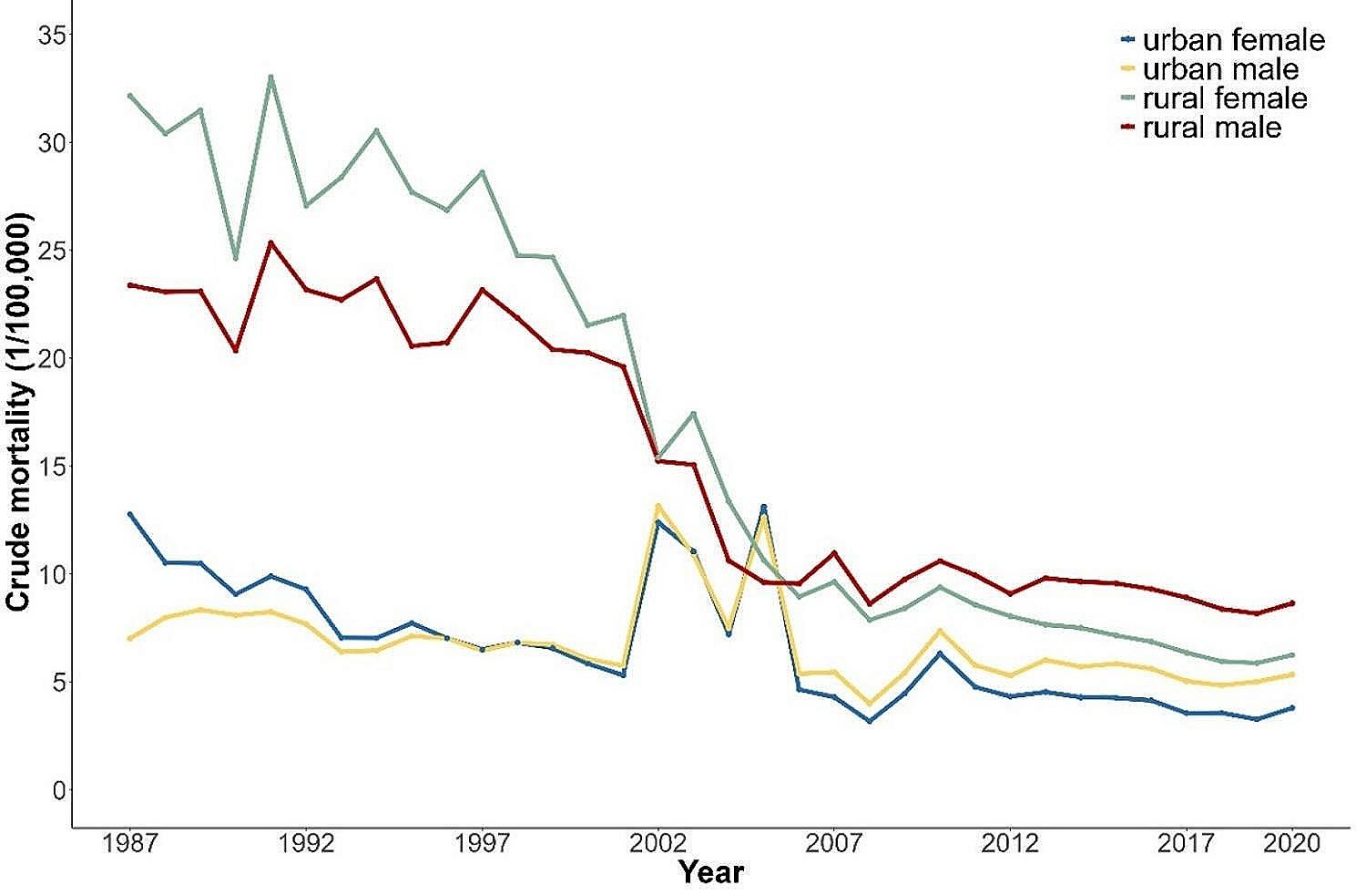
Trends in crude suicide mortality in urban and rural China by sex: 1987–2020

**Fig. 2:**
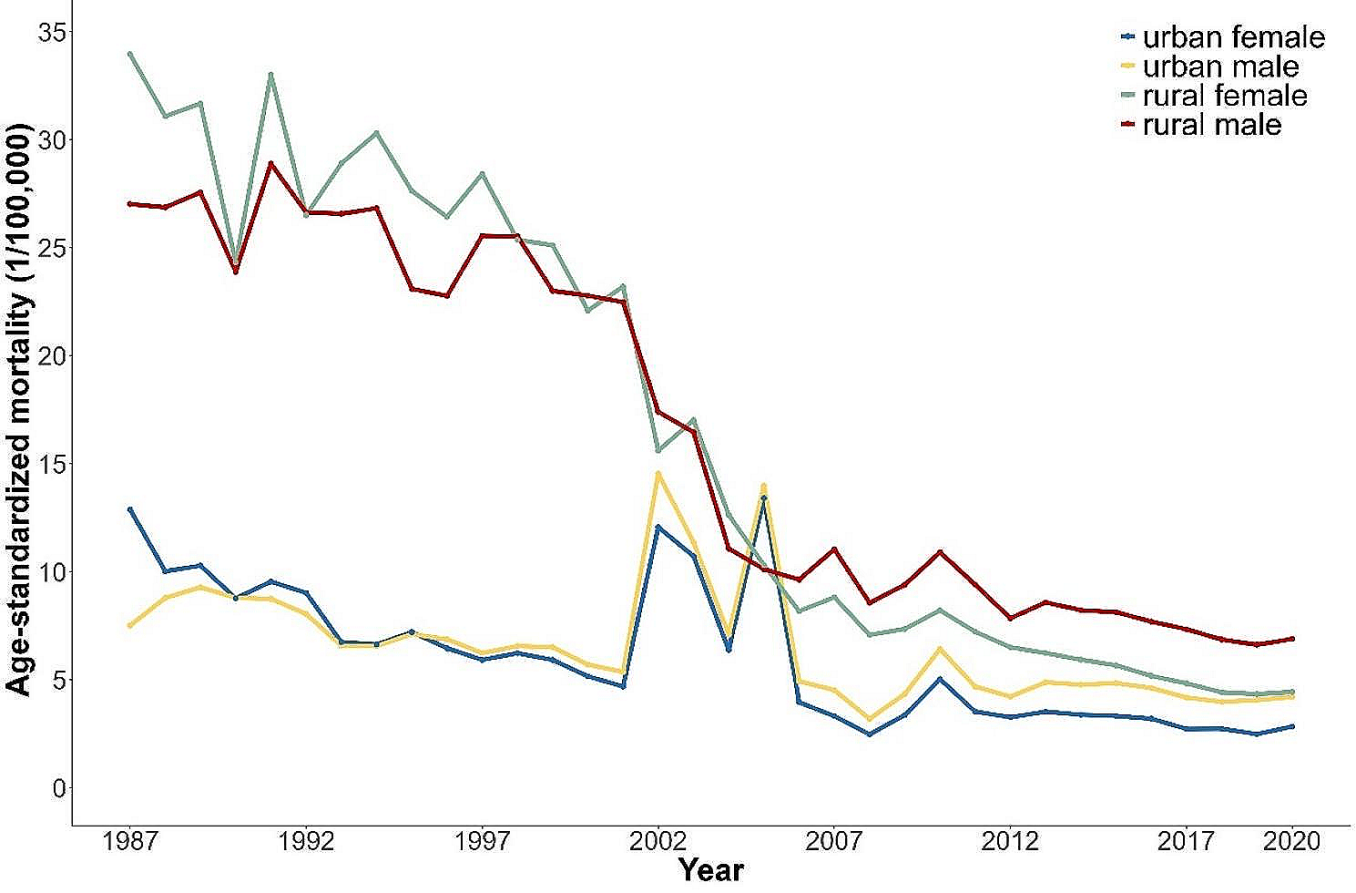
Trends in age-standardized suicide mortality in urban and rural China by sex: 1987–2020. **Note**: The data were standardized by World Standard Population

Figures 1 and 2 shows the long-term trends of crude and age-standardized suicide mortality rates in China from 1987 to 2020. During the study period, both crude and age-standardized suicide mortality rates exhibited a similar downward trend. The urban age-standardized suicide mortality rate dropped roughly 66%, while the rural equivalent decreased by nearly 81%. In rural areas, male and female suicide mortalities were downward from 1987 to 2020. Females had higher mortality than males before 2005. Post-2005, this gender mortality gap subtly reversed and has been gradually widening. In contrast, urban suicide mortality rates have been decreasing at a slower pace, with two notable periods of fluctuation between 2001–2004 and 2004–2006. A same gender reversal in urban suicide mortality rates occurred around 1996.

### Phase change in suicide mortality based on Joinpoint regression analysis

**Fig. 3:**
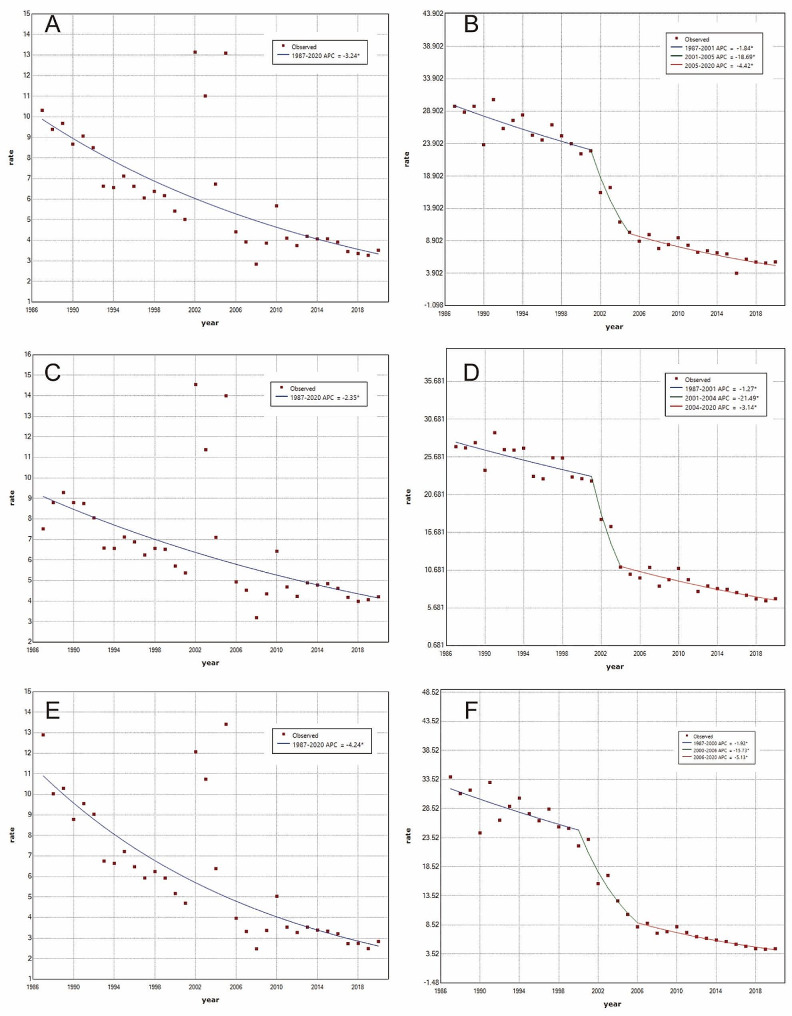
Joinpoint analysis of age-standardized suicide mortality in urban and rural areas. **Note**: Joinpoint analysis of age-standardized suicide mortality in urban total population **(A)**, rural total population **(B)**, urban males **(C)**, rural males **(D)**, urban females **(E)**, and rural females **(F)**

The findings concerning Joinpoint analysis are shown in Table 1; Fig. 3. Age-standardized suicide mortality in urban areas decreased consistently from 1987 to 2020 (average annual percent change (AAPC) = -3.2%, *p* < 0.05). The age-standardized suicide mortality rates for urban males and females followed similar downtrends, with females’ AAPC of -4.2% and males’ AAPC of -2.3%. In contrast, the age-standardized suicide mortality in rural areas declined at a sharper rate (AAPC = -5.2%, *p* < 0.05), but this decline was not linear. Instead, it fell into three distinct stages: 1987–2001, 2001–2005, and 2005–2020, transitioning from a steady decrease to an accelerated drop, before settling into a steady downtrend again. In addition, the decline in the age-standardized suicide mortality was roughly synchronized for rural males and females.

**Table 1:** Joinpoint analysis of age-standardized suicide mortality in urban and rural areas

Residence	Mortality Rate ^a^ (per 100,000)				Entire Range ^b^			Segment 1				Segment 2				Segment 3		
		1987	2020		AAPC (%)	95% CI		Period	APC (%)	95% CI		Period	APC (%)	95% CI		Period	APC (%)	95% CI
Urban	Total	10.31	3.52		-3.2*	(-4.2,-2.3)		1987–2020	-3.2*	(-4.2,-2.3)		NA	NA	NA		NA	NA	NA
	Male	7.51	4.20		-2.3*	(-3.3,-1.4)		1987–2020	-2.3*	(-3.3,-1.4)		NA	NA	NA		NA	NA	NA
	Female	12.89	2.84		-4.2*	(-5.3,-3.2)		1987–2020	-4.2*	(-5.3,-3.2)		NA	NA	NA		NA	NA	NA
Rural	Total	29.65	5.64		-5.2*	(-7.3,-3.1)		1987–2001	-1.8*	(-3.4,-0.2)		2001–2005	-18.7*	(-31.7,-3.3)		2005–2020	-4.4*	(-5.8,-3.0)
	Male	27.03	6.89		-4.2*	(-6.0,-2.4)		1987–2001	-1.3*	(-2.2,-0.3)		2001–2004	-21.5*	(-36.1,-3.6)		2004–2020	-3.1*	(-3.9,-2.4)
	Female	33.96	4.44		-5.9*	(-7.0,-4.9)		1987–2000	-1.9*	(-3.1,-0.7)		2000–2006	-15.7*	(-20.1,-11.2)		2006–2020	-5.1*	(-6.2,-4.1)

**Note**. (a) Standardized by the World Standard Population from the World Health Organization; (b) The period from 1987 to 2020; Abbreviations: APC = annual percent change; AAPC = average annual percent change; NA = Not Applicable, referring to no join-points identified; * = Significantly difference from zero (*p* < 0.05).

### Net drift and local drift in suicide mortality from 1987 to 2020

**Fig. 4:**
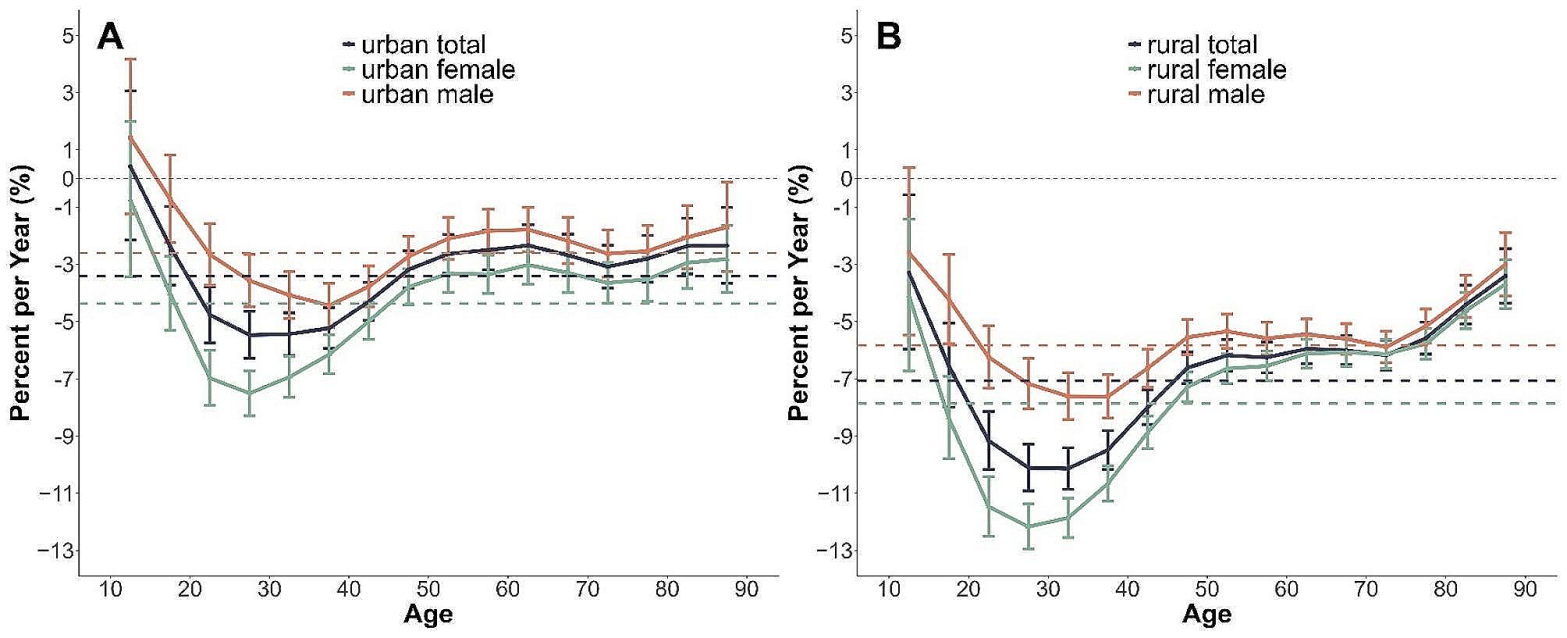
Local drift with net drift values for suicide mortality and sex difference by area in China from 1987 to 2020. **Note.** Net drift represents the overall annual percentage change, and the values were all < 0, indicating substantial reductions in suicide mortality across the study period. Local drift values represent annual percentage change in each age group, and most of the local drift values were also < 0 in both sexes, indicating a decreased trend in suicide mortality across the study period; this figure considered 16 5-year age groups (from 10–14 years group to 85 + years group)

The net drift and local drift values represented the annual percentage changes in the expected age-standardized and age-specific suicide mortality rates, respectively (Fig. 4, Table S1). During the study period, there was a general annual net drift of -3.41% (95% confidence interval (CI), -3.77% to -3.05%, *p* < 0.01) in urban areas and − 7.07% (95% CI, -7.38% to -6.75%, *p* < 0.01) in rural areas. Both of these represent significant changes, as they did not fall within − 1% to 1% per year. Gender-based differences in the yearly trends of age-standardized suicide mortality rates were also pronounced. The decline was quicker for women than for men in both urban (-4.37% versus − 2.6%) and rural (-7.86% versus − 5.83%) settings. Furthermore, local drift charts indicated that nearly all age groups in both urban and rural settings had local drift values below zero. Urban and rural local drift trends display a sustained U-shape, with the lowest point of the curve at the 20–29 age group, indicative of the steepest decline rate. The decline rate in age-specific suicide mortality lessened consistently with age in the 30–59 and 75–89 age groups and leveled off for the 60–74 age group. Notably, all local drift values in rural areas were mostly lower than those in urban areas.

### Age-period-cohort effects on suicide mortality

**Fig. 5:**
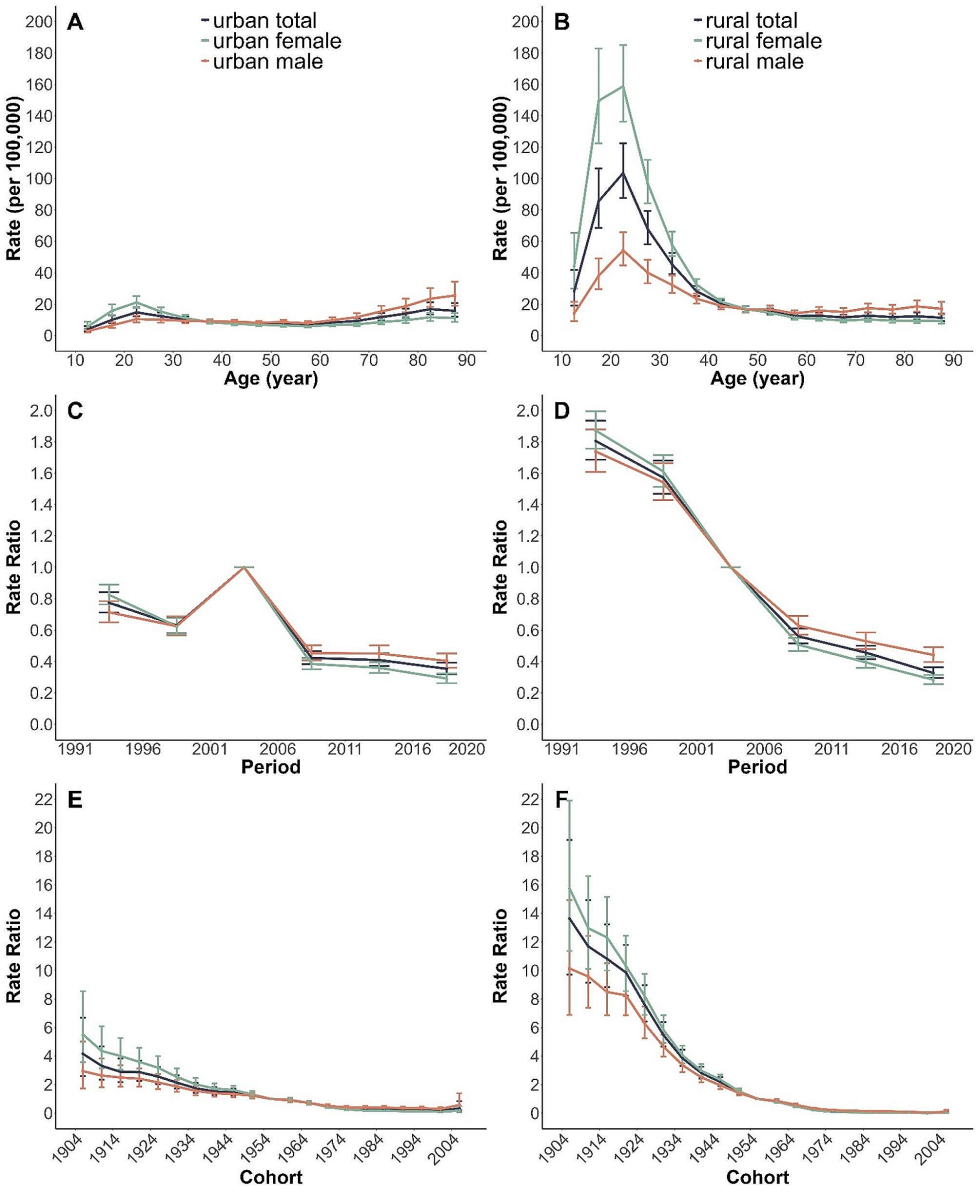
Parameter estimates of age, period, and cohort effects on suicide mortality in China from 1987 to 2020. **Note**: **(A)** Age effects on mortalities in urban area; **(B)** Age effects on mortalities in rural area; **(C)** Period effects on mortalities in urban area **(D)** Period effects on mortalities in rural area; **(E)** Cohort effects on mortalities in urban area; **(F)** Cohort effects on mortalities in rural area

Figure 5 shows estimates of age, period, and cohort effects on suicide. In terms of age effects, both rural and urban locations observe a peak in suicide age-specific mortality within the 20–24 age group of the same birth cohort. Notably, suicide age-specific mortality rates are higher for females than males before the age of 45 but the trend reverses afterwards. There is considerable variation in the age effect between rural and urban areas. Primarily, rural residents aged 10–44, specifically those within the 15–34 age group, indicate a considerably elevated suicide risk compared to their urban counterparts. The risk is the highest in rural females aged 20–29. Conversely, urban and rural groups aged over 50 exhibit distinct behavioral patterns. Urbanites show an increasing suicide risk with age, with the gender gap progressively broadening. Nonetheless, this widening gender disparity is not evident and remains largely stable in rural areas. In terms of period effects, the period rate ratios were found to have similar monotonic decreasing patterns for both genders in rural areas during the whole period. However, for urban areas, although the suicide risk also showed a downward trend over the whole period, there were marked fluctuations between period 1996–2000 and period 2001–2005. The drop in period effects is more distinct in rural regions as compared to urban ones. Additionally, both genders across urban and rural environments display a monotonic decline in cohort rate ratios. The reduction pace in rural and female groups surpassed that of their opposite counterparts in all the successive cohorts.

### Sensitivity analysis

The sensitivity analysis using GBD data revealed that both crude and age-standardized suicide mortalities exhibited a declining trend in both genders, which were initially higher in females but later surpassed by males (Figure S1-S2). It aligns with our results. In the age-period-cohort analysis (Figure S3-S4), the local drift curves, and the trends observed in age and cohort effects of suicide mortality were consistent with our curve trends. Regarding period effects, the results from GBD were closely mirrored the rural pattern in our study, but differed lightly from the urban period effects pattern, which shows an initial upward trend followed by a subsequent decline. Overall, the results obtained using GBD data are almost consistent with our main findings.

The sensitivity analysis results, where the mortality rates of specific years were replaced with the means, are shown in Figure S5-S6. The suicide mortality trends for rural males and females remained consistent with the original results, while the abnormal increase in suicide mortality rates among urban males and females in 2005 disappeared. However, there still existed a noticeable elevated phase post-2002, consistent with the original findings.

Additionally, the sensitivity analysis results for adjusting suicide underreporting are shown in Figure S7-S10. The overall trends of suicide mortality crude rates and standardized rates remain almost consistent with the original findings, except for a slight advancement in the years of early cross-over between male and female mortality rates. The age-period-cohort model results indicate that, compared to the unadjusted data, the trend of increasing suicide mortality rates with age among urban elderly population becomes more pronounced, while others show no significant changes. Overall, the core outcomes remain almost consistent before and after correcting suicide underreporting.

## Discussion

Based on the Joinpoint regression and age-period-cohort framework, this study provided the first comprehensive overview of long-term patterns in suicide mortality in China from 1987 to 2020, highlighting the disparities between urban and rural regions. From a holistic perspective, both crude and age-standardized suicide mortality rates in China experienced a persistent decline during these years, including in urban and rural areas, with the suicide risk of rural groups declining faster. The downward trend in the suicide rate may reflect the effect of economic improvement, urbanization, and the consequent much more limited household access to pesticides in China. However, the recent slower decrease in suicide mortality also reminded us to guard against a possible suicide backlash. In terms of specific demographic categories, suicide mortality rates continued to diverge based on factors such as age, gender, and rural-urban disparity, albeit in new patterns. For one, youngsters aged 20–29 and the elderly were at higher suicide risk in the whole population, especially individuals aged 20–24. Moreover, females had a higher suicide risk than males, but there existed a trend reversal between genders in groups above 45. Remarkably, rural populations aged 10–45, especially females of reproductive age, carried a substantially higher suicide risk than their urban counterparts. Both period and cohort effects regarding suicide rates experienced steeper declines in rural regions as compared to urban areas. Lastly, a notable difference in suicide risk trends was observed among urban and rural populations over the age of 50 – while the gap in suicide risk between males and females widened with age in urban areas, this was not the case in rural regions. These findings could provide certain reference for future suicide prevention efforts in China.

Our study’s findings, demonstrating a reduction in China’s overall suicide mortality, aligns with previous research [6, 7, 9, 10]. In contrast to countries such as America, Japan, and South Korea [28, 29], China’s suicide mortality rate has been on the decline for over thirty years. Earlier research implies that this reduction could be attributed to several socioeconomic factors including economic growth, rapid urbanization, improved medical emergency systems and transportation, enhanced healthcare accessibility, and reduced availability of lethal pesticides in rural areas [30–33]. Since 1987, China’s urbanization has significantly increased, reaching a rate of 45.4% according to China’s seventh national census [34]. With the lower suicide mortality in urban areas, the urbanization process seems to have positively contributed to declines in suicide mortality by providing better emergency medical systems and healthcare accessibility. Moreover, since 2000, China has witnessed a gradual decline in the unemployment rate among its labor force, which might have contributed positively to the overall reduction in the country’s suicide rate on a macroscopic scale [35]. Previous studies have shown that rising unemployment rates may lead to health detriments, consequently heightening the risk of suicide [36–38]. However, the downward trend in suicide mortality in rural communities has been slower since 2006. This could be primarily attributable to the escalating aging population [39], increased workforce migration [40], the issue of left-behind children and the elderly in rural areas [39, 41] and the global economic recession [42, 43]. These challenges could impede the rapid decrease in suicide mortality in China. Given that China’s suicide mortality rate is already relatively low globally, it is plausible that the current trend may persist.

Age stands as a substantial demographic factor influencing suicide rates. The varying risk associated with different age groups necessitates tailored prevention and intervention strategies. This study underscores that both the youth (aged 20–24) and the elderly are two significant high-risk groups for suicide within China. Past research conducted in South America [44], Europe [45, 46], and Asia [47, 48], has consistently shown that those over 50 years of age possess the greatest risk. Factors such as chronic illness, family discord, financial difficulty, and mental disorders may contribute significantly to the suicide rates among the elderly in China [49, 50]. One study also proposes that the decrease in suicide rates among the elderly may be attributable to enhanced healthcare systems and a reduction in poverty rates [51]. Moreover, it’s critical to note that age-period-cohort studies, predominantly outside China, have not identified young adults aged 20–24 as the highest suicide risk group. This discrepancy suggests a unique circumstance within China. The causes for increased risk in this age group remain uncertain. In China’s unique exam-oriented context, significant physiological and psychological changes occur between the ages of 20 and 24. If not adequately addressed, the sudden increase in life and work stress would likely contribute to substantial psychological problems. The reason may partly explain the high suicide mortality in this age group. In the future, we need to pay extra attention to high-risk age groups above, and adopt targeted suicide prevention and intervention strategies.

The urban-rural disparity in suicide mortality in China was continuing marked but showed new changing patterns. The primary shift is seen among rural females of childbearing age, who historically had higher suicide risk levels and decline rates compared to their urban counterparts. Prior elevated suicide risk in this demographic was likely attributed to their unfavorable economic conditions, low social status, dependency on family and males, high occupational stress, and lack of adequate medical emergency services for impulsive suicide attempts [10, 52, 53]. However, increased socio-economic status, improved living conditions, and better access to healthcare have greatly reduced the suicide risk among rural women [10]. Further contributing to this decline are restrictions on pesticides and a significant rural-urban migration of women [53, 54]. Second, there were gender trend reversals in suicide risks among urban and rural population aged 50 years and older. Specifically, after age 50, male suicide rates surpass female rates, a finding consistent with previous research [55]. The gender gap in suicide risk widens with age in urban males and females, but remains relatively stable within their rural counterparts. It’s hypothesized that this trend could be attributed to the faster urban life pace, a heightened sense of self-loss, and diminished family connections among urban retirees, underscoring the need for mental health attention in the wake of increasing urbanization. Finally, while both the period and cohort effects on suicide rates have decreased more in rural areas than urban ones, the patterns of urban and rural suicide rates are converging. This probably stems mainly from China’s urban-rural integration and increased attention to suicide issues over the past 30 years. There is a need for further research to inform targeted suicide intervention strategies, taking evolving urban-rural disparities into account.

This study also has several limitations that require much attention. First, the change of death category coding in 2002 and the alteration of urban-rural definitions in 2005 may introduce bias to the suicide mortality data. Despite obtaining consistent results through mean imputation, we cannot fully eliminate the impact of this issue. The related findings need to be interpreted cautiously. Second, the mortality data in the early years only covered approximately 10% of China’s population, with survey samples mainly from regions with well-established reporting mechanisms, such as eastern and central cities [4]. The geographical imbalance in the sources of mortality data, inconsistent standards in data quality control, and underreporting of suicide deaths may affect the authenticity of suicide data and introduce bias in temporal analysis [4, 14]. Although we cannot completely eliminate the inherent limitations of these early data sources, we conducted sensitivity analyses using GBD data from different sources and the adjusted data considering underreporting effects, and obtained conclusions similar to those of this study. Within the currently rather limited framework of accessible suicide data, these analyses could provide a diversified perspective. Third, the huge rural-urban migration population and the method of attributing deaths based on residence registration booklets and the location of death may confound urban and rural mortality rates, requiring careful consideration of their impact on the conclusions of this study. Lastly, like other age-period-cohort analyses, this study is susceptible to potential ecological fallacies. The results derived at the population level could not accurately reflect individual scenarios. Thus, insights gleaned from the age-period-cohort analysis in this study require further validation in future individual-based studies.

## Conclusion

Suicide mortality rates in China continued to decline from 1987 to 2020. Despite this, significant disparities persist along the lines of age, gender, and urban-rural location and new patterns are emerging. Females of childbearing age and the elderly are at high risk for suicide. The slower decrease, high-risk groups, and the reversing urban-rural gender trends urgently require close attention and more targeted suicide prevention programs.

### Electronic supplementary material

Below is the link to the electronic supplementary material.

Supplementary Material 1

## Data Availability

The data sets generated or analyzed within the scope of this study are accessible in the “China Health Statistics Yearbook”, a repository maintained by the National Health and Health Commission of China. (https://pan.baidu.com/s/11enIs8I_c_Ml-v-lKvSw8A?pwd=uf8w)
