# Supplementary material for: The long-term changing patterns of suicide mortality in China from 1987 to 2020: continuing urban-rural disparity
Source: BMC Public Health. 2024 May 9;24:1269. doi: 10.1186/s12889-024-18743-z (PMC11083847; doi:10.1186/s12889-024-18743-z)
Supplement: Supplementary file 1 — Supplementary Material 1 [file 12889_2024_18743_MOESM1_ESM.docx]

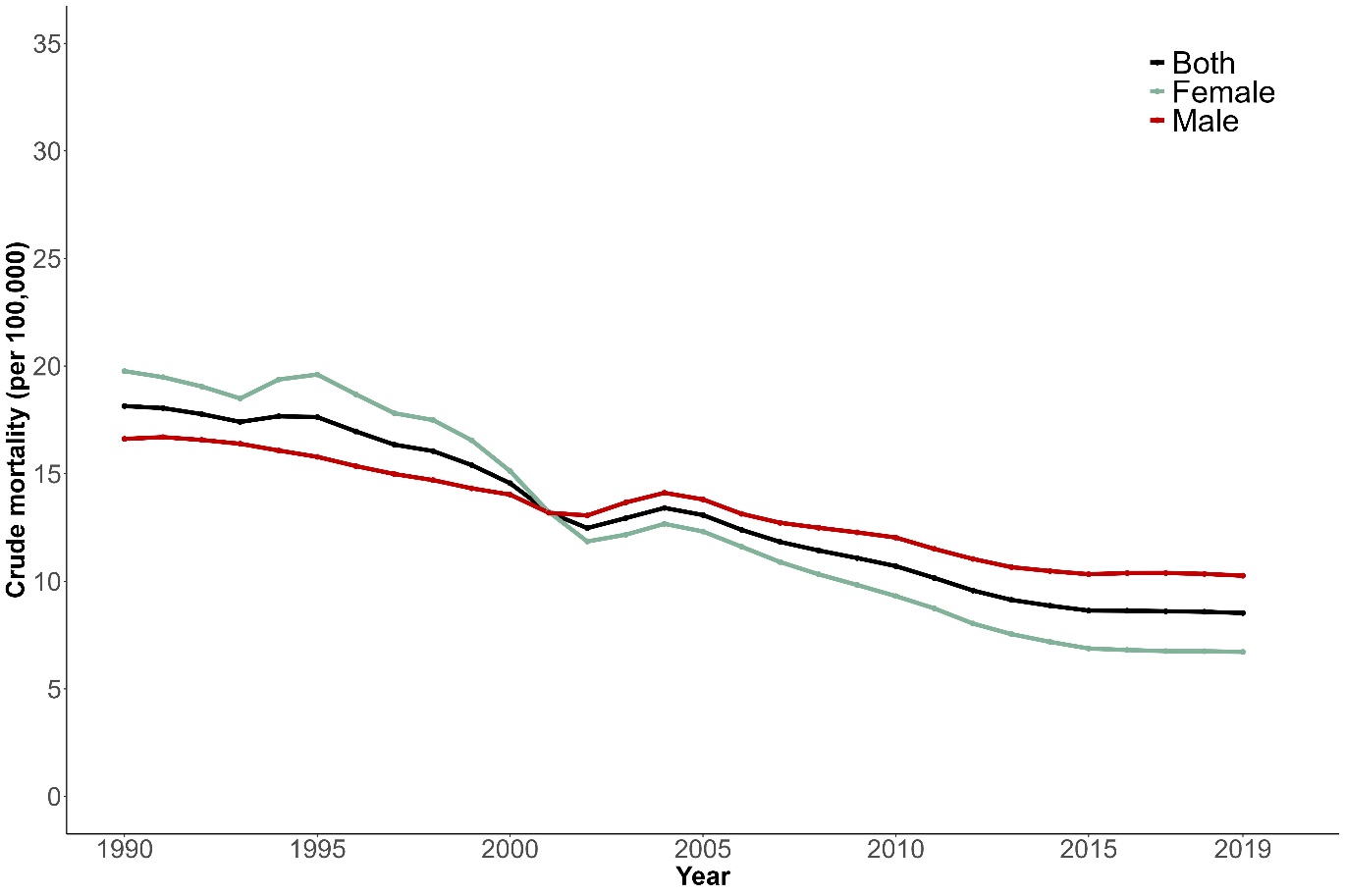


**Figure S1. Trends in crude suicide mortality in China by sex: 1990-2019, using GBD data.**


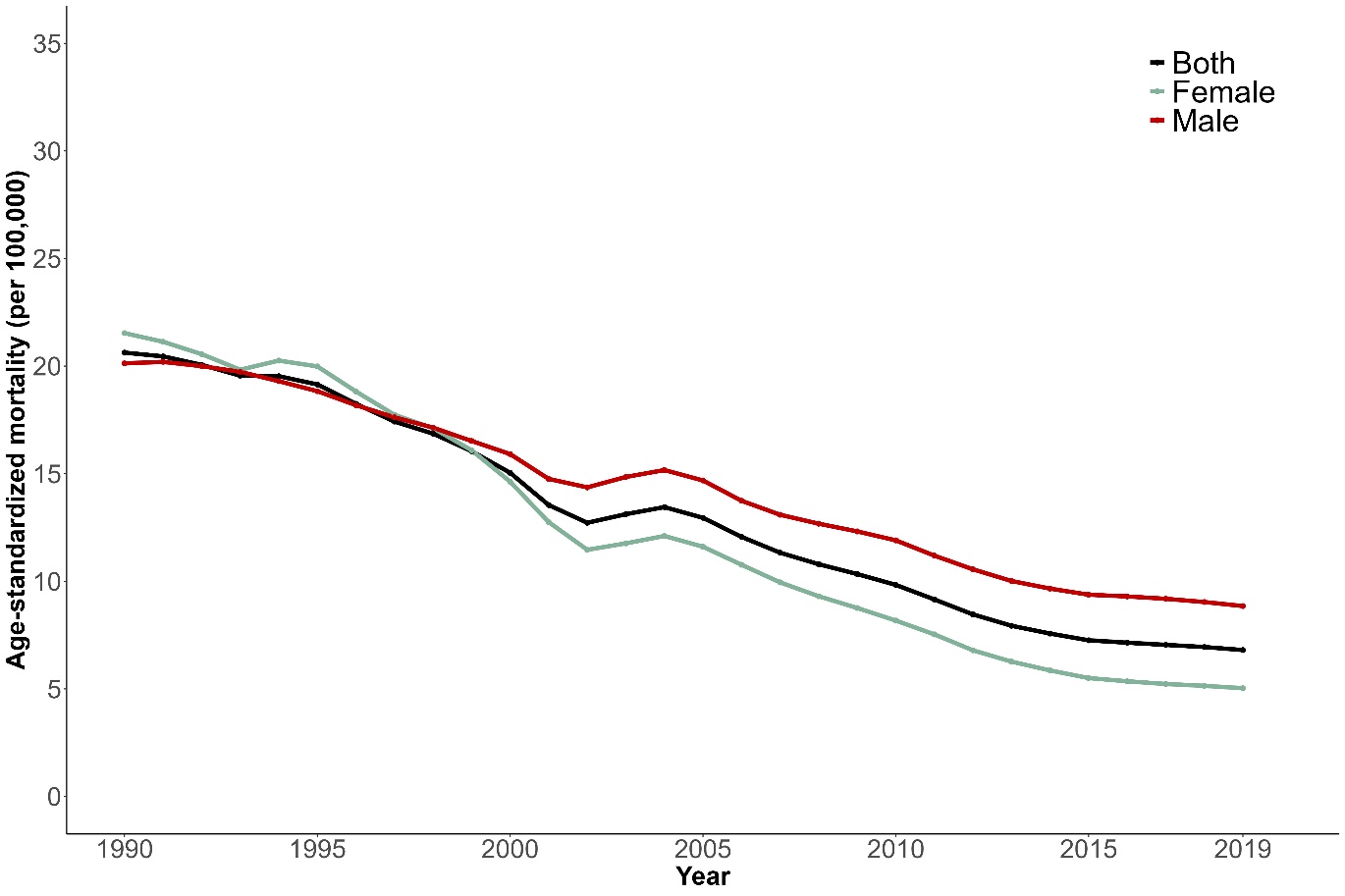


**Figure S2. Trends in age-standardized suicide mortality in China by sex: 1990-2019, using GBD data.**

**Note:** The data were standardized by GBD Standard Population.


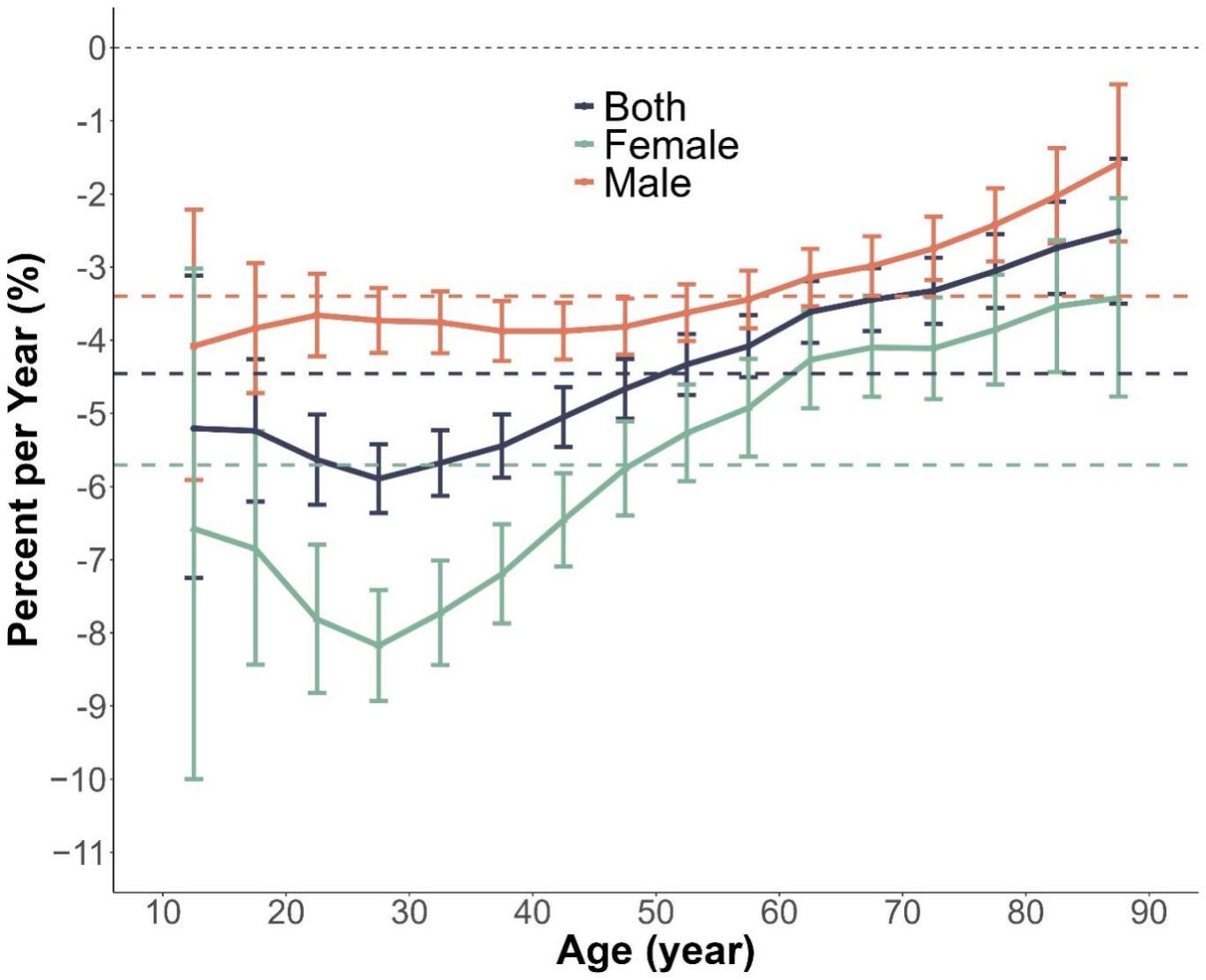


**Figure S3. Local drift with net drift values for suicide mortality and sex difference by area in China from 1990 to 2019, using GBD data.**

**Note:** this figure considered 16 5-year age groups (from 10-14 years group to 85+ years group)


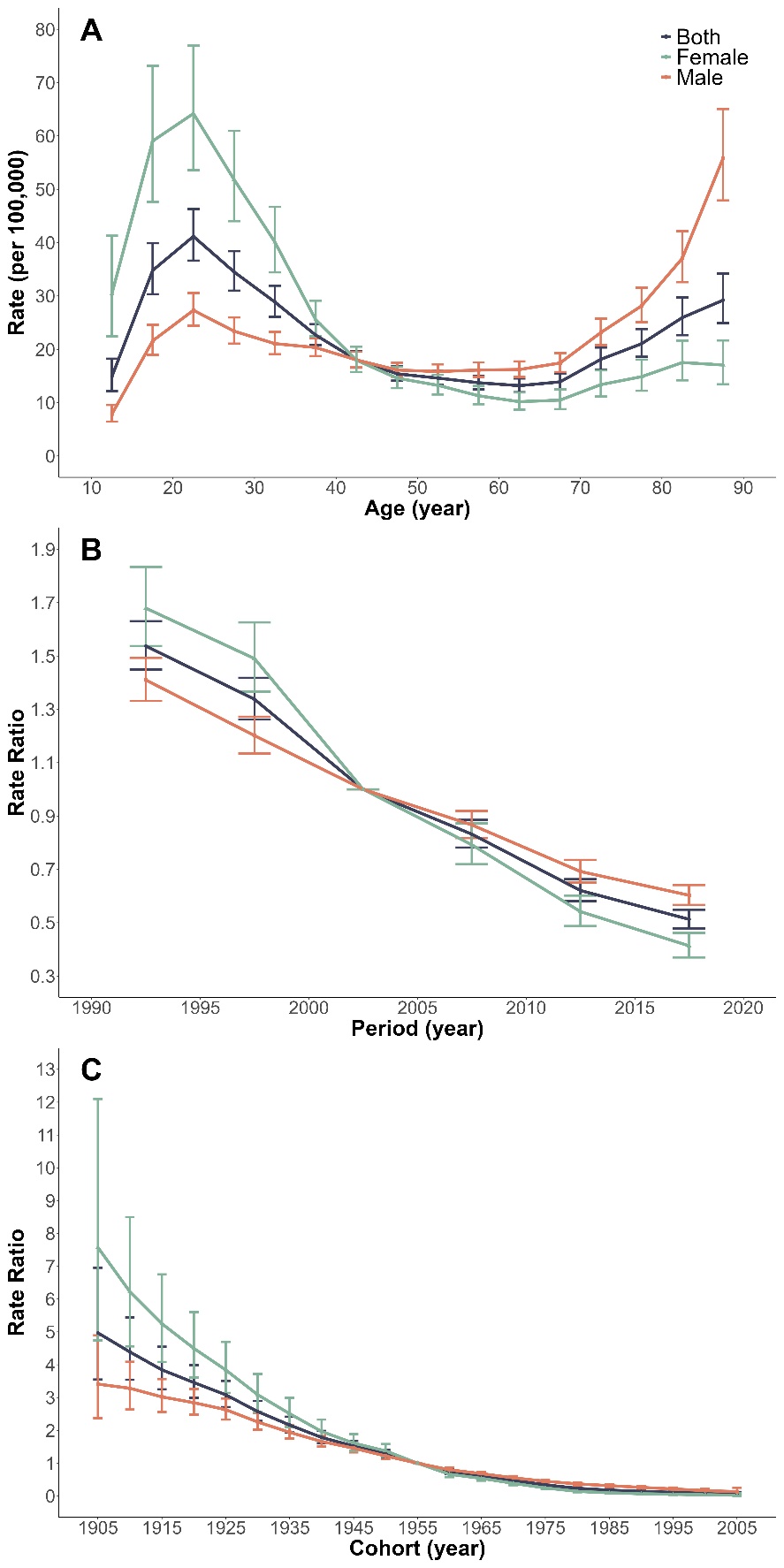


**Figure S4. Parameter estimates of age, period, and cohort effects on suicide mortality in China from 1990 to 2019, using GBD data.**

**Note:** (A) Age effects; (B) Period effect; (C) Cohort effects


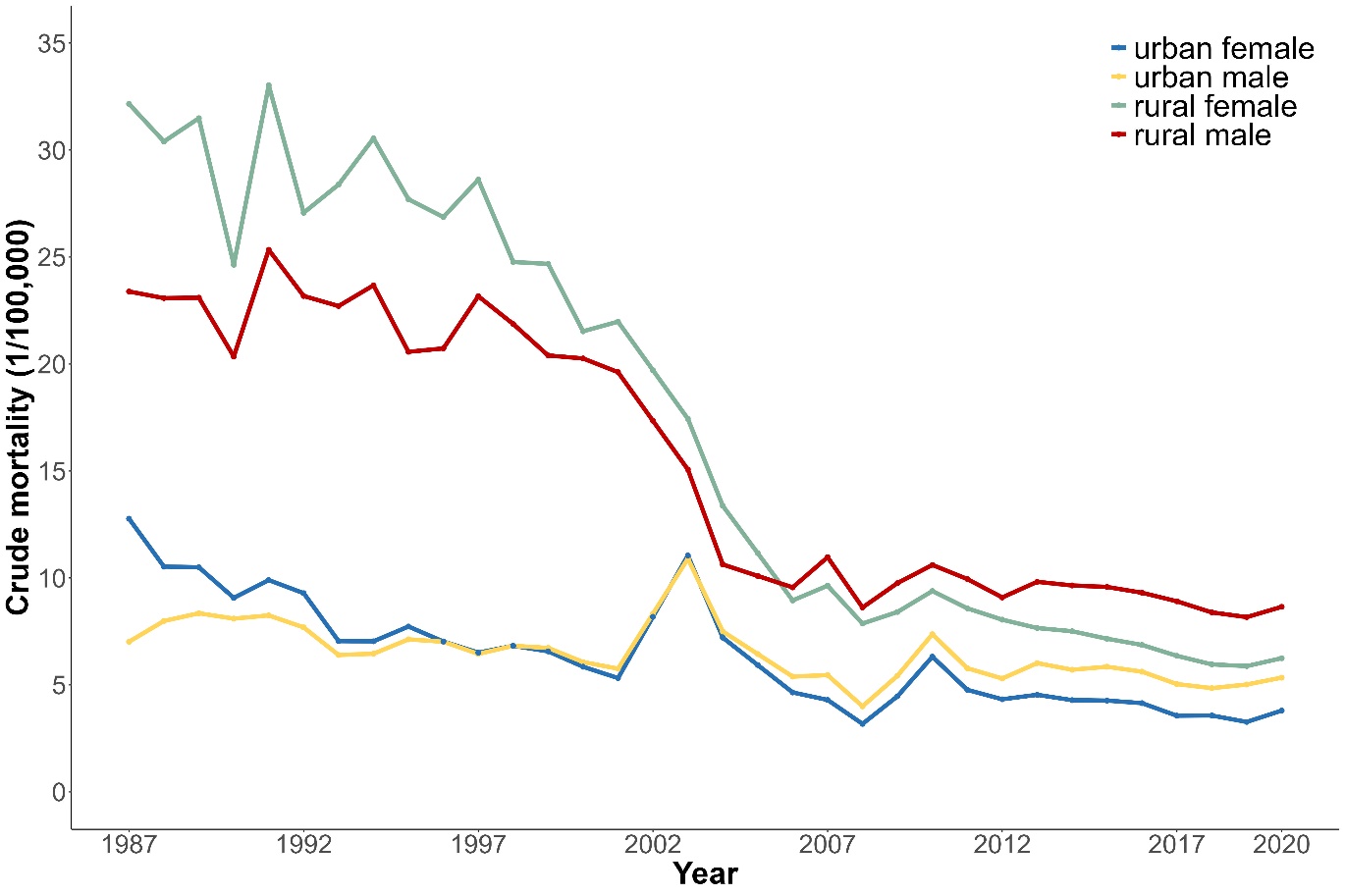


**Figure S5. Trends in crude suicide mortality in urban and rural China by sex: 1987-2020, with mortalities of 2002 and 2005 replaced.**


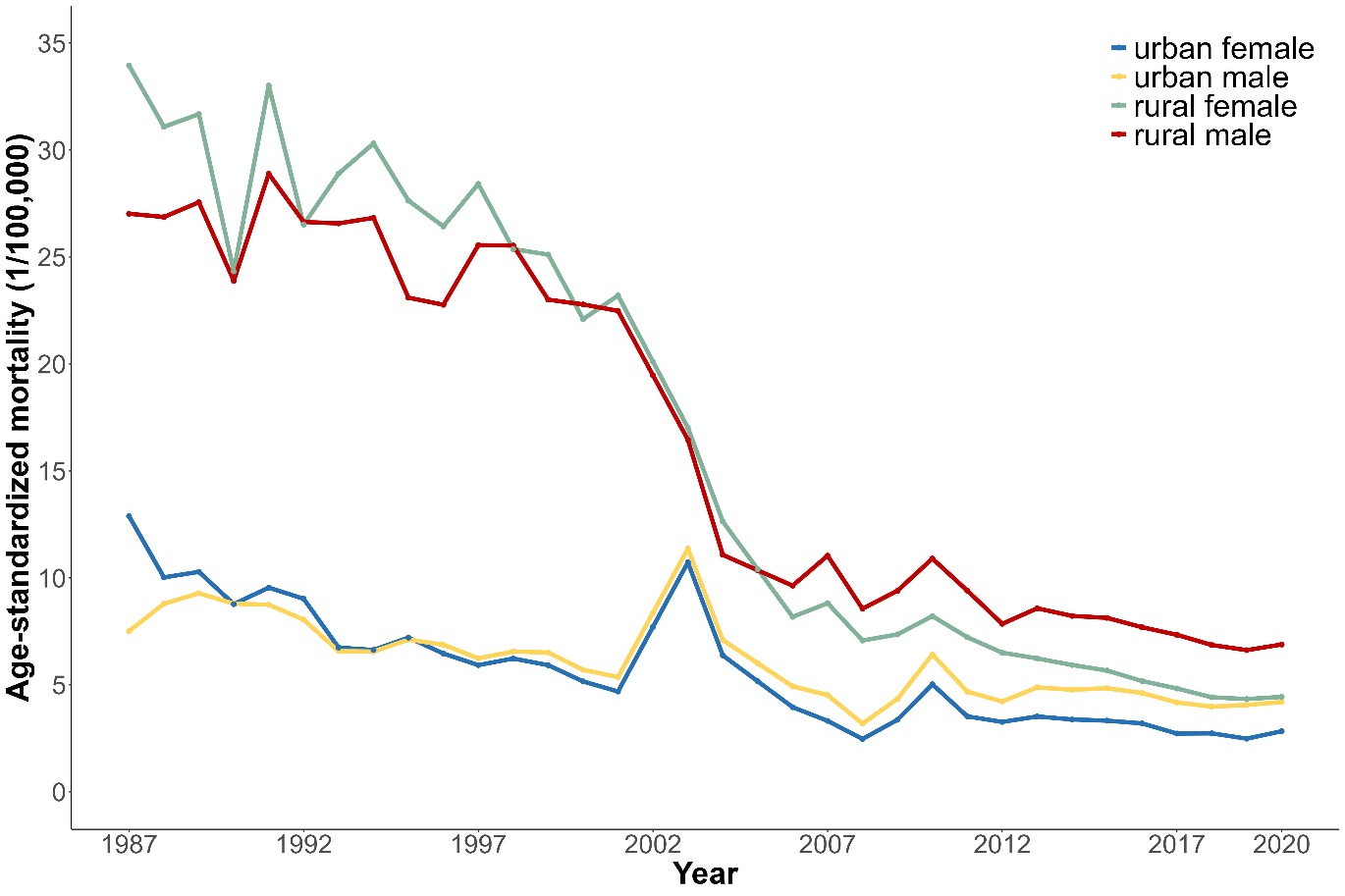


**Figure S6. Trends in age-standardized suicide mortality of in urban and rural China by sex: 1987-2020, with mortalities of 2002 and 2005 replaced.**

**Note:** The data were standardized by World Standard Population.

**
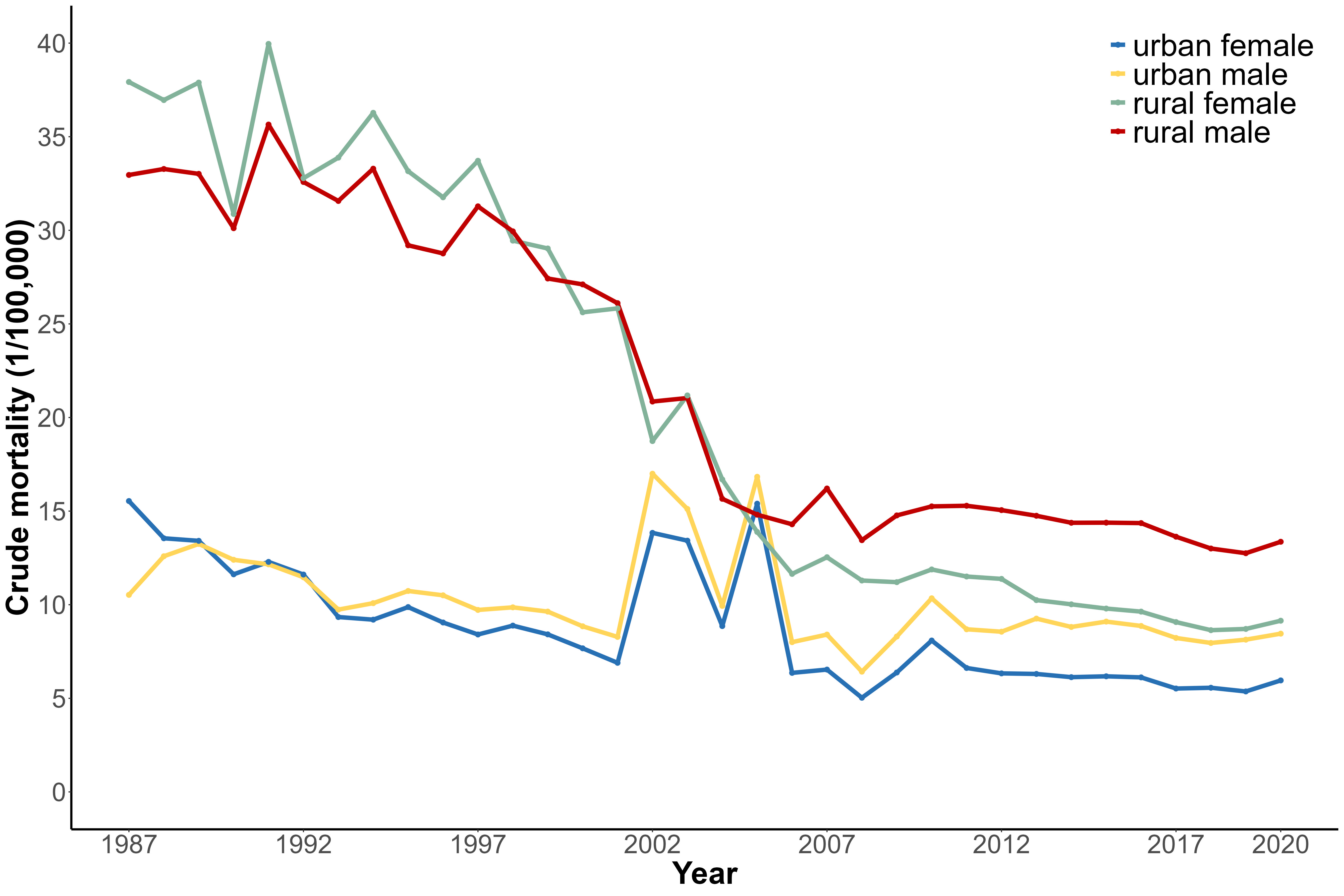
**

**Figure S7. Trends in crude suicide mortality in urban and rural China by sex: 1987-2020, adjusting for mortality underreporting rates.**

**
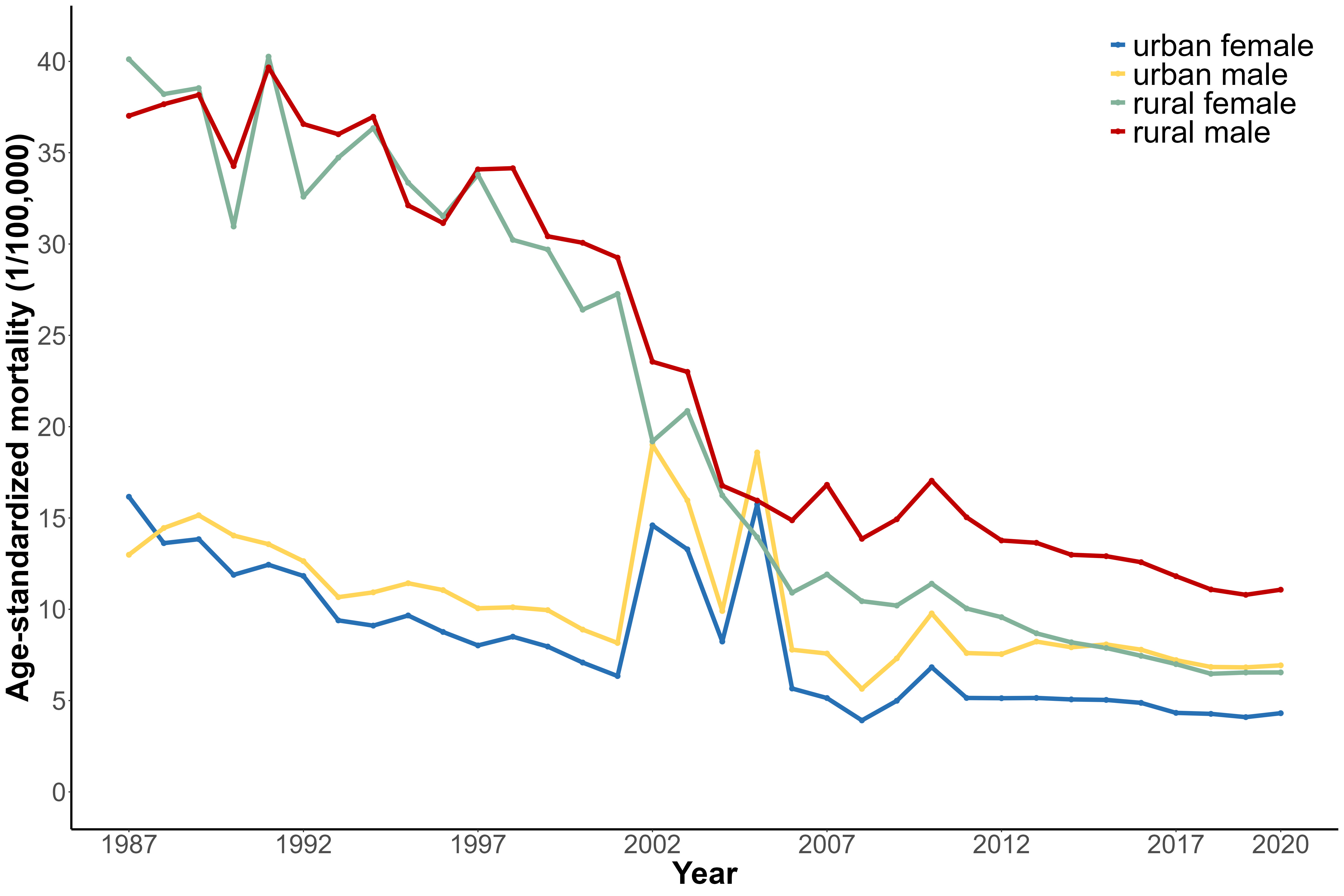
**

**Figure S8. Trends in age-standardized suicide mortality of in urban and rural China**

**by sex: 1987-2020, adjusting for mortality underreporting rates.**

**Note:** The data were standardized by World Standard Population.

**
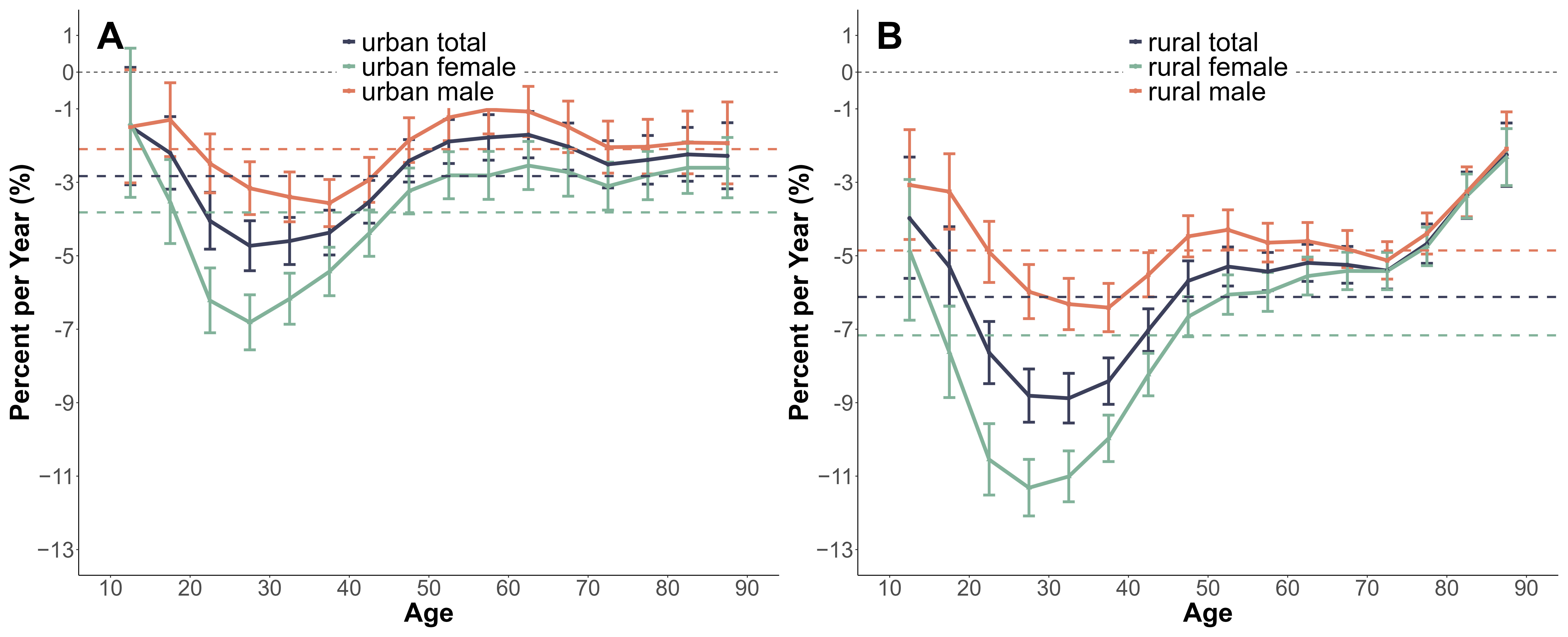
**

**Figure S9. Local drift with net drift values for suicide mortality and sex difference by area in China from 1987 to 2020, adjusting for mortality underreporting rates.**

**Note.** Net drift represents the overall annual percentage change, and the values were all <0, indicating substantial reductions in suicide mortality across the study period. Local drift values represent annual percentage change in each age group, and most of the local drift values were also <0 in both sexes, indicating a decreased trend in suicide mortality across the study period; this figure considered 16 5-year age groups (from 10-14 years group to 85+ years group).

**

**

**Figure S10. Parameter estimates of age, period, and cohort effects on suicide mortality in China from 1987 to 2020, adjusting for mortality underreporting rates.**

**Note:** (A) Age effects on mortalities in urban area; (B) Age effects on mortalities in rural area; (C) Period effects on mortalities in urban area (D) Period effects on mortalities in rural area; (E) Cohort effects on mortalities in urban area; (F) Cohort effects on mortalities in rural area.

**Table S1. Local drift with net drift values for suicide mortality and sex difference by area in China from 1987 to 2020.**

| Residence and gender | Age group/Net | Local drift/Net drift (%) | | |
| --- | --- | --- | --- | --- |
|  |  | Percent per Year | Lower 95% CI | Upper 95% CI |
| Urban total | 10-14 | 0.42 | -2.15 | 3.07 |
|  | 15-19 | -2.37 | -3.73 | -0.99 |
|  | 20-24 | -4.76 | -5.74 | -3.78 |
|  | 25-29 | -5.47 | -6.28 | -4.65 |
|  | 30-34 | -5.44 | -6.18 | -4.69 |
|  | 35-39 | -5.23 | -5.93 | -4.52 |
|  | 40-44 | -4.30 | -4.96 | -3.63 |
|  | 45-49 | -3.18 | -3.83 | -2.52 |
|  | 50-54 | -2.63 | -3.30 | -1.95 |
|  | 55-59 | -2.50 | -3.20 | -1.80 |
|  | 60-64 | -2.34 | -3.05 | -1.62 |
|  | 65-69 | -2.68 | -3.41 | -1.94 |
|  | 70-74 | -3.08 | -3.83 | -2.32 |
|  | 75-79 | -2.80 | -3.62 | -1.98 |
|  | 80-84 | -2.36 | -3.33 | -1.38 |
|  | 85+ | -2.35 | -3.67 | -1.01 |
|  | Net | -3.41 | -3.77 | -3.05 |
| Urban male | 10-14 | 1.43 | -1.25 | 4.18 |
|  | 15-19 | -0.73 | -2.25 | 0.82 |
|  | 20-24 | -2.66 | -3.73 | -1.57 |
|  | 25-29 | -3.57 | -4.48 | -2.66 |
|  | 30-34 | -4.08 | -4.90 | -3.25 |
|  | 35-39 | -4.44 | -5.20 | -3.66 |
|  | 40-44 | -3.78 | -4.50 | -3.05 |
|  | 45-49 | -2.72 | -3.43 | -2.01 |
|  | 50-54 | -2.10 | -2.83 | -1.36 |
|  | 55-59 | -1.83 | -2.59 | -1.07 |
|  | 60-64 | -1.79 | -2.57 | -1.00 |
|  | 65-69 | -2.18 | -2.98 | -1.36 |
|  | 70-74 | -2.63 | -3.45 | -1.79 |
|  | 75-79 | -2.54 | -3.43 | -1.64 |
|  | 80-84 | -2.05 | -3.14 | -0.95 |
|  | 85+ | -1.70 | -3.25 | -0.13 |
|  | Net | -2.60 | -2.99 | -2.22 |
| Urban female | 10-14 | -0.75 | -3.43 | 2.00 |
|  | 15-19 | -4.02 | -5.32 | -2.71 |
|  | 20-24 | -6.98 | -7.94 | -6.01 |
|  | 25-29 | -7.51 | -8.29 | -6.72 |
|  | 30-34 | -6.94 | -7.65 | -6.22 |
|  | 35-39 | -6.15 | -6.82 | -5.47 |
|  | 40-44 | -4.98 | -5.63 | -4.33 |
|  | 45-49 | -3.79 | -4.43 | -3.15 |
|  | 50-54 | -3.32 | -3.98 | -2.66 |
|  | 55-59 | -3.35 | -4.02 | -2.68 |
|  | 60-64 | -3.02 | -3.70 | -2.33 |
|  | 65-69 | -3.29 | -3.99 | -2.59 |
|  | 70-74 | -3.65 | -4.36 | -2.93 |
|  | 75-79 | -3.53 | -4.29 | -2.76 |
|  | 80-84 | -2.94 | -3.84 | -2.04 |
|  | 85+ | -2.82 | -3.98 | -1.64 |
|  | Net | -4.37 | -4.71 | -4.02 |
| Rural total | 10-14 | -3.30 | -5.96 | -0.56 |
|  | 15-19 | -6.54 | -8.00 | -5.06 |
|  | 20-24 | -9.16 | -10.18 | -8.14 |
|  | 25-29 | -10.11 | -10.91 | -9.30 |
|  | 30-34 | -10.14 | -10.87 | -9.41 |
|  | 35-39 | -9.49 | -10.16 | -8.82 |
|  | 40-44 | -8.00 | -8.60 | -7.40 |
|  | 45-49 | -6.62 | -7.18 | -6.05 |
|  | 50-54 | -6.18 | -6.73 | -5.63 |
|  | 55-59 | -6.25 | -6.78 | -5.72 |
|  | 60-64 | -5.95 | -6.47 | -5.44 |
|  | 65-69 | -6.01 | -6.52 | -5.49 |
|  | 70-74 | -6.18 | -6.70 | -5.66 |
|  | 75-79 | -5.58 | -6.14 | -5.02 |
|  | 80-84 | -4.41 | -5.08 | -3.74 |
|  | 85+ | -3.40 | -4.35 | -2.45 |
|  | Net | -7.07 | -7.38 | -6.75 |
| Rural male | 10-14 | -2.59 | -5.48 | 0.38 |
|  | 15-19 | -4.22 | -5.77 | -2.64 |
|  | 20-24 | -6.24 | -7.32 | -5.15 |
|  | 25-29 | -7.17 | -8.06 | -6.28 |
|  | 30-34 | -7.61 | -8.42 | -6.80 |
|  | 35-39 | -7.62 | -8.37 | -6.86 |
|  | 40-44 | -6.63 | -7.31 | -5.96 |
|  | 45-49 | -5.55 | -6.17 | -4.93 |
|  | 50-54 | -5.34 | -5.93 | -4.75 |
|  | 55-59 | -5.58 | -6.15 | -5.02 |
|  | 60-64 | -5.45 | -5.99 | -4.91 |
|  | 65-69 | -5.61 | -6.14 | -5.07 |
|  | 70-74 | -5.89 | -6.43 | -5.35 |
|  | 75-79 | -5.16 | -5.76 | -4.56 |
|  | 80-84 | -4.12 | -4.85 | -3.39 |
|  | 85+ | -3.00 | -4.10 | -1.88 |
|  | Net | -5.83 | -6.17 | -5.50 |
| Rural female | 10-14 | -4.10 | -6.72 | -1.41 |
|  | 15-19 | -8.37 | -9.79 | -6.92 |
|  | 20-24 | -11.47 | -12.50 | -10.42 |
|  | 25-29 | -12.17 | -12.95 | -11.38 |
|  | 30-34 | -11.86 | -12.55 | -11.17 |
|  | 35-39 | -10.67 | -11.29 | -10.04 |
|  | 40-44 | -8.88 | -9.44 | -8.31 |
|  | 45-49 | -7.28 | -7.81 | -6.75 |
|  | 50-54 | -6.64 | -7.16 | -6.12 |
|  | 55-59 | -6.55 | -7.07 | -6.04 |
|  | 60-64 | -6.12 | -6.62 | -5.61 |
|  | 65-69 | -6.07 | -6.57 | -5.57 |
|  | 70-74 | -6.14 | -6.65 | -5.63 |
|  | 75-79 | -5.77 | -6.31 | -5.23 |
|  | 80-84 | -4.62 | -5.26 | -3.99 |
|  | 85+ | -3.69 | -4.54 | -2.84 |
|  | Net | -7.86 | -8.17 | -7.55 |
